# β-Hydroxy-β-Methylbutyrate (HMB) Normalizes Dexamethasone-Induced Autophagy-Lysosomal Pathway in Skeletal Muscle

**DOI:** 10.1371/journal.pone.0117520

**Published:** 2015-02-06

**Authors:** María D. Girón, Jose D. Vílchez, Sathyavageeswaran Shreeram, Rafael Salto, Manuel Manzano, Elena Cabrera, Nefertiti Campos, Neile K. Edens, Ricardo Rueda, Jose M. López-Pedrosa

**Affiliations:** 1 Department of Biochemistry and Molecular Biology II, University of Granada, Granada, Spain; 2 Abbott Nutrition R&D, Singapore, Singapore; 3 Abbott Nutrition R&D, Granada, Spain; 4 Abbott Nutrition R&D, Columbus, Ohio, United States of America; University of Louisville School of Medicine, UNITED STATES

## Abstract

Dexamethasone-induced muscle atrophy is due to an increase in protein breakdown and a decrease in protein synthesis, associated with an over-stimulation of the autophagy-lysosomal pathway. These effects are mediated by alterations in IGF-1 and PI3K/Akt signaling. In this study, we have investigated the effects of β-Hydroxy-β-methylbutyrate (HMB) on the regulation of autophagy and proteosomal systems. Rats were treated during 21 days with dexamethasone as a model of muscle atrophy. Co-administration of HMB attenuated the effects promoted by dexamethasone. HMB ameliorated the loss in body weight, lean mass and the reduction of the muscle fiber cross-sectional area (shrinkage) in gastrocnemius muscle. Consequently, HMB produced an improvement in muscle strength in the dexamethasone-treated rats. To elucidate the molecular mechanisms responsible for these effects, rat L6 myotubes were used. In these cells, HMB significantly attenuated lysosomal proteolysis induced by dexamethasone by normalizing the changes observed in autophagosome formation, LC3 II, p62 and Bnip3 expression after dexamethasone treatment. HMB effects were mediated by an increase in FoxO3a phosphorylation and concomitant decrease in FoxO transcriptional activity. The HMB effect was due to the restoration of Akt signaling diminished by dexamethasone treatment. Moreover, HMB was also involved in the regulation of the activity of ubiquitin and expression of MurF1 and Atrogin-1, components of the proteasome system that are activated or up-regulated by dexamethasone. In conclusion, *in vivo* and *in vitro* studies suggest that HMB exerts protective effects against dexamethasone-induced muscle atrophy by normalizing the Akt/FoxO axis that controls autophagy and ubiquitin proteolysis.

## Introduction

Muscle atrophy occurs in various conditions such as fasting or disuse as well as in diseases including cancer, diabetes, AIDS, sepsis, denervation or glucocorticoid treatment. It happens when proteolysis exceeds protein synthesis, which leads to a reduction of muscle fiber cross-sectional area and a decrease in muscle strength [1]. In muscle cells, proteolysis is essentially mediated by the activity of two highly conserved and independent systems, the autophagic-lysosomal and the ubiquitin-proteasomal pathways [2–4].

Autophagy is a physiological process utilized by skeletal muscle to sequester cytoplasmic proteins and organelles into vacuoles known as autophagosomes. These fuse with lysosomes, leading to the digestion of the contents by lysosomal hydrolases [3]. Autophagy is vital for removing aged and damaged cellular components, the breakdown of undedicated nutrient stores, and the remodeling of cellular architecture. However, examination of various skeletal muscle disorders has shown that the autophagic system is over-stimulated in different conditions leading to muscle atrophy [3,4]. Upregulation of autophagy and lysosomal genes has been documented at the transcript and protein level (microtubule-associated protein I light chain 3 (LC3), p62 and Bnip3) in atrophying muscles [2].

In the ubiquitin proteasome system, most soluble and myofibrilar muscle proteins are targeted for degradation through attachment of ubiquitin molecules. The ubiquitin ligases, Atrogin-1 and MURF1, are markedly induced during early states of muscle atrophy [4–7]. Interestingly, the rate of muscle atrophy is markedly reduced by targeted inactivation of these genes [8]. These pathways are regulated by specific transcription factors, such as Forkhead box O (FoxO) [4,9,10] which are normally phosphorylated and inactivated by Akt in the cytosol, rendering them unable to enter the nucleus [5,6].

Glucocorticoids have been used in *in vitro* [10] and *in vivo* [7] models of muscle atrophy. The signaling pathway stimulated by glucocorticoids involved a decrease in protein synthesis and an upregulation of atrogenes, in part through a reduction in PI3K/Akt signaling and a concomitant increase in FoxO activity [2,5]. In rats, treatment with IGF-1 reverted the upregulation of autophagy-related genes and ubiquitin proteasome elements induced by glucocorticoids [6].

β-Hydroxy-β-methylbutyrate (HMB) is a leucine metabolite. Positive effects of HMB on muscle protein turnover in animal models of muscle wasting due to sepsis, age or disuse have been described [11–13]. Experiments carried out in cell cultures have proposed that HMB effects are mediated via the MAPK/ERK and PI3K/Akt pathways [14,15] and have focused mainly on the proteasomal pathway [16]. However, no effort has been made to study the effects of HMB on the autophagic-lysosomal pathway in muscle.

In the present study, we have investigated the effects of HMB on a rat model of muscle wasting induced by DEX. In this model HMB exerts positive effects on lean body mass recovery and functionality. In a well-established *in vitro* model of muscle atrophy induced by DEX, we studied the effects of HMB on the autophagic-lysosomal and ubiquitin proteasome pathways. The main finding indicates that HMB could contribute to preventing the deleterious effects of glucocorticoids by targeting FoxO, as the key player that orchestrated not only ubiquitin proteasomal system but also the autophagic-lysosomal pathway. In fact, for the first time, our results point to the involvement of HMB in minimizing muscle protein degradation through modulation of the autophagic-lysosomal system. Taken together, these results support a new mechanism in which HMB may prevent glucocorticoid-induced muscle atrophy.

## Materials and Methods

### Materials

HMB and DEX were purchased from Sigma (St. Louis, MO, USA). HMB calcium salt (Ca-HMB) was from Technical Sourcing International, Inc., Missoula, MT (purity 98.5%). Tissue culture media, Fetal Bovine Serum (FBS) and supplements were from Sigma. ERK1/2 and phospho-ERK1/2 E10 (Thr202/Tyr204), PKB/Akt and phospho-PKB/Akt (Ser473), S6K1 and phospho-S6K1 (Thr389) antibodies were from Cell Signaling Technology (Beverly, MA, USA). FKHRL1 (FoxO3A), p-FKHRL1 (FoxO3a) (Thr32), MuRF1 and SQSTM1 (p62) antibodies were from Santa Cruz Biotechnology (Santa Cruz, CA, USA). FBXO32 (Atrogin-1) antibody was from Acris Antibodies GmbH (Herford, Germany). LC3 antibody was from MBL International Corporation (Buckingham, UK). The Cignal FoxO Reporter (luc) kit (CCS-1022L) was from SABiosciences (Quiagen Inc. Valencia, California). Plasmid pGFP-LC3 was kindly provided by Professor T. Yoshimori (Department of Genetics, Osaka University), Addgene plasmid #21073. HRP-conjugated secondary antibodies were from BIO-RAD (Madrid, Spain).

### Animals

Male Sprague Dawley rats (8~10 weeks-old) weighing 237.5 ± 2.5 g were housed in standard plastic cages under controlled environmental conditions (22 ± 3°C temperature, 50 ± 20% humidity, a light/dark cycle of 12 hours each and 15–20 fresh air changes per hour). Animals were housed group-wise (2–3 animals per cage) and were fed *ad libitum* a certified Irradiated Laboratory Rodent Diet (Nutrilab brand, Tetragon Chemie Pvt. Ltd., Bangalore). All the animals were kept under acclimatization for a period of about 5–7 days before initiation of the experiment.

Three experimental groups were defined: Control (n = 6), DEX (n = 7) and HMB/DEX (n = 7). Ca-HMB to the HMB/DEX group was administered in drinking water (320 mg/kg/BW) [13] from 7 days prior to DEX treatment and mantained during the full time-course of the experiment. DEX was administered intraperitoneally once a day in a dose of 0.1mg/kg body mass. A control group received saline only. Initiation of DEX injection was considered as day 0 and treatment was carried out for 21 days. Body weight and food intake were recorded daily until the end of the study. On day 21, all the animals were sacrificed with carbon dioxide euthanasia.

### Ethics Statement

This study was conducted in accordance with the regulations of the Committee for the Purpose of Control and Supervision of Experiments on Animals (CPCSEA), Government of India and Association for Assessment and Accreditation of Laboratory Animal Care (AAALAC) compliance. The ‘Form B’ for carrying out animal experimentation was reviewed and approved by the Institutional Animal Ethics Committee (SYNGENE/IAEC/286/03-2012). All aspects of the protocol and experimental design, including animal number, anesthesia and euthanasia methodologies were provided.

### Body composition analysis

Body composition analyses were performed using ECHO-MRI (Model no 900, EchoMRI LLC, Houston, TX 77079).

### Histochemistry and cross-sectional area measurements

At the end of the experiment, rats were sacrificed with carbon dioxide euthanasia. Soleus and gastrocnemius muscles were weighed. Unilateral gastrocnemius was preserved in 10% neutral buffered formalin for muscle fiber morphometric analysis.

After tissue fixation, processing and embedding, muscles were cut into 5–7 μm thick transverse sections using a microtome (Microm, HM340E Germany) and mounted on glass slides. Samples were stained with hematoxylin and eosin (H&E) using an autostainer (Leica ST5010, Germany). Transverse sections of muscle were randomly selected and photographed using light microscopy (Nikon Eclipse 80i, Japan) with a digital camera (Leica DFC425C, Germany). Six microscopic fields per muscle were analyzed at 40x magnification. The surface area of individual muscle bundles was measured and expressed as μm2 using “Leica Application Suit” analysis bundle. Cross-sectional areas were determined for at least 300 muscle fibers per animal. The areas with sectioning artifacts and edges were excluded while taking photomicrographs.

### Functionality measures

The grip strength test was used as a measure of limb strength. In this procedure, the grip strength meter (Model—0271–004 M, Columbus Instruments, USA) was set in tension mode and tared before each measurement [17].

### Cell culture

The L6.C11 rat skeletal muscle myoblast line (ECACC No. 92102119) was grown in DMEM supplemented with 10% (v/v) FBS, 2 mmol/l glutamine plus 100 units/ml penicillin and 0.1 mg/ml streptomycin in an atmosphere of 5% CO2 and 95% air, and was maintained at sub-confluent densities in the growth media. Cells were differentiated into myotubes by culturing them for 5 days in DMEM containing 2% FBS (v/v).

### Determination of protein synthesis

Protein synthesis was measured as described by Gulve et al. [18] with the following modifications. L6 cells were plated in 48-well tissue culture plates and differentiated for 5 days. Cells were treated with 25 μM HMB for two hours in DMEM with 10% FBS and 0.8 mM L-Tyrosine and then for 1 h with 5 μM DEX in the absence or presence of effectors. Cells were then spiked with 1 μCi/ml of L-[ring-3,5–3H]-Tyrosine (Perkin Elmer, Waltham MA) and were incubated for 1 h. The reaction was stopped by placing the plates on ice. All wells were thoroughly washed twice with ice cold PBS media containing 2 mM non-radioactive L-Tyrosine (PBS-Tyr) and the cells then lysed in 0.1 ml of 0.1 mM NaOH/0.1% sodium deoxycholate. The cellular proteins were precipitated by adding 0.1 ml of cold 20% trichloroacetic acid (TCA; Sigma). This mixture was then incubated at 4°C for 15 min. After centrifugation (16,000×g for 10 min at 4°C), the pellet was washed once with cold 10% TCA and then, the precipitated proteins were dissolved in 0.1 ml of 1 M NaOH. An aliquot (5 μl) of the NaOH-solubilized material was used for total protein quantitation and the remaining dissolved proteins were neutralized with 1 M HCl, mixed with ReadySafe scintillation fluid (Beckman Coulter, Brea CA) and radiolabel determined with a scintillation counter (Beckman Coulter). Data was computed as dpm/μg of proteins.

### Protein degradation

Myotubes (5 days in differentiation media) were labeled with 1 μCi/ml of L-[ring-3,5–3H]-Tyrosine for 48 h in DMEM plus 10% FBS in presence or absence of 25 μM HMB. Cells were rinsed once in PBS-Tyr and then placed in DMEM supplemented with 10% FBS, 2 mM L-tyrosine plus 5 μM DEX (degradation medium) for 2 h to allow degradation of very short-lived proteins. The cells were then rinsed twice in PBS-Tyr and fresh degradation medium was added. Cells were incubated for an additional 24 h in degradation medium. Degradation rates were determined as previously described [19].

### Autophagy Assay

pGFP-LC3-expressing L6 myotubes were used to assess autophagy. L6 myoblasts were transiently transfected with pGFP-LC3 vector and then differentiated into myotubes for 3 days. Cells were used at 80–90% confluence and transfection was performed using LipofectAMINE 2000 (Invitrogen). 5 hours later the transfection reagent was removed and fresh medium was added to the cultures. In the case of cultures set to differentiate into myotubes, differentiation medium was added 24 h later and during 3–5 days. L6 transfected myotubes were pre-incubated for 30 min with 25 μM HMB and then incubated for 30 min with 5 μM DEX in the presence or absence of effectors. 100 nM Bafilomycin A1 was added concomitantly with the effectors and remained in the culture throughout the treatment. Cells were fixed with 2% paraformaldehyde in PBS for 15 minutes at room temperature and coverslips were mounted on glass slides using Vectashield mounting media (Vector Laboratories, Inc., Burlingame, CA). Confocal microscopy was performed on a Leica DMI6000 confocal microscope. eGFP was excited using the 488 nm line of a krypton/argon laser and the emitted fluorescence was detected with a 504–562 nm channel. All samples were exposed to laser for a time interval not >5 min to avoid photobleaching. The laser was set to the lowest power able to produce a fluorescent signal. Maximum voltage of photomultipliers was used to decrease the required laser power as much as possible. A pinhole of 1 Airy unit was used. Images were acquired at a resolution of 1024 x 1024. Series were acquired in the xyz mode. Data was processed using Leica AF software package. Autophagosomes were quantified by counting GFP-LC3-positive dots.

### Protein phosphorylation analysis

To study the phosphorylation status of the proteins involved in signaling events, L6 myotubes were incubated with 25 μM HMB for 1h and then treated in the absence or presence of 5 μM DEX for 30 min. Plates were flash frozen in liquid nitrogen and processed as described previously [20,21].

To study the expression of proteins associated with protein degradation, L6 myotubes were pre-incubated for 48 h with 25 μM HMB and then incubated for 24 h with 5 μM DEX in the presence or absence of effectors. When 100 nM Bafilomycin A1 was used, it was added concomitantly with DEX and remained in the culture throughout the treatment. After treatment, they were lysed with RIPA buffer supplemented with phosphatase and protease inhibitors, 10 mM sodium fluoride, 10 mM sodium pyrophosphate, 1 mM sodium orthovanadate, 1 mM EGTA, 20 nM okadaic acid, 10 μg/ml aprotinin, 10 μg/ml leupeptin and 10 μg/ml pepstatin.

The protein concentration was measured using the bicinchoninic acid method [22]. Proteins (40 μg) were separated by SDS-PAGE, transferred onto nitrocellulose membranes, and immunoblotted with specific antibodies; the immunoblots were developed by an enhanced chemiluminescence detection method.

### RNA Purification and Retrotranscription

Total RNA was isolated from L6 myotubes using the GeneJet RNA Purification Kit (Thermo Scientific). The amount of total RNA was measured using a NanoVue Plus Spectrophotometer (GE Healthcare Life Sciences) and the quality was verified by 1% agarose gel electrophoresis. 1 μg of total RNA from each sample was reverse-transcribed using Maxima First Strand cDNA Synthesis Kit for RT-qPCR (Thermo Scientific).

### Quantitative Real-time PCR (qPCR)

The qPCR was performed using specific primers on a MiniOpticon Real-Time PCR System (Bio-Rad) with Bio-Rad CFX Manager 3.1 software and fluorescence signal detection (SYBR Green) after each amplification cycle. Sequences of the primers were for β-actin, forward 5’-AAGACCTCTATGCCAACAC-3’ and reverse 5’-TGATCTTCATGGTGCTAGG-3’; Bnip3, forward 5’- CTACTCTCAGCATGAGAAAC-3’ and reverse 5’-TCCAATGTAGATCCCCAATC-3’; murf (Trim63), forward 5’-GTTTACTGAAGAGGAGGAGG-3’ and reverse 5’- AGAAGACACACTTCCCTATG-3’; Atrogin-1 (Fbxo32), forward 5’-TACAACTGAACATCATGCAG-3’ and reverse 5’- GTACATCTTCTTCCAATCCAG-3’. First strand cDNA was used as a template in 10 μl reactions including 5 μl of 2x iTaq Universal SYBR Green supermix and 500 nM of each primer. Negative controls (with no DNA template) for each primer set were included in each run. qPCR cycling was performed at 95°C (2 min), followed by 40 cycles at 95°C (15 s), 57°C (30 s), and, finally, a melt curve program (60–95°C with a heating rate of 0.1°C/s and a continuous fluorescence measurement). Measurements of gene expression with regard to each RNA extraction were obtained in triplicate. Relative expression of mRNA was calculated using the 2−ΔCt method with β-actin as an internal reference gene.

### Reporter gene constructs and assays

For the analysis of rat Ubiquitin (UbC) promoter, a 417 bp fragment flanking the transcription start point was amplified from rat genomic DNA. This fragment is similar to those characterized for human and rat promoters [20]. Sequences of all constructs were verified by automated DNA sequencing. The Cignal FoxO Reporter (luc) kit contains a mixture of inducible FoxO-responsive firefly luciferase construct and constitutively expressing Renilla luciferase construct (40:1).

For gene reporter analysis, cells were used at 80–90% confluence. Transfection was performed using LipofectAMINE2000 as described above. The DNA mixture comprised the pGL3-UbC luciferase reporter and the reference plasmid pRL-TK (ratio 95:5) or the Cignal FoxO Reporter mixture.

L6 myotubes were incubated in presence or absence of 25 μM HMB for 48 h. Cells were then incubated in the presence or absence of 5 μM DEX for 24 h. Luciferase activity was determined using the Dual Luciferase method (Promega) in a luminometer (Sirius L, Berthold Technologies, Bad Wildbad, Germany) and results were standardized for Renilla luciferase activity. To allow comparison of the expression patterns, the data are expressed as relative changes in luciferase activity and were normalized to a value of 100%.

### Statistics

Results are expressed as mean ± SEM. Statistical analysis was performed by one way ANOVA followed by Tukey test as appropriate. P < 0.05 was considered statistically significant.

## Results and Discussion

Many pathological states characterized by muscle atrophy are associated with an increase in glucocorticoid levels [7,10]. The enhancement of muscle proteolysis by glucocorticoids results from a decrease in the PI3K/Akt signaling and the concomitant activation of the ubiquitin proteasome and lysosomal autophagy systems [7].

In muscle atrophy, the altered protein turnover is due mainly to an increase in the rate of protein breakdown. This process is mediated by an induction of the ubiquitin proteasome system, involving an increase in ubiquitin activity and ubitiquin ligase levels [4–7].

Autophagy is a physiological house-keeping process involved in protein and organelle degradation with cell-protective functions [23]. However, defective regulation of autophagy leads to myopathy. Thus, tight control of the autophagic flux must be mantained in order to preserve muscle mass and prevent wasting [24].

Ubiquitin-Proteasome activity and autophagy have been described as likely to proceed in an independently coordinated manner [5,6]. This could be explained by the existence of a common transcription factor that orchestrates muscle atrophy by controlling both pathways. In fact, FoxO has been identified as the master regulator of both processes in skeletal muscle [9].

In the present study we have shown that HMB exerts protective effects against muscle atrophy promoted by DEX *in vivo* and *in vitro*. In our model of muscle atrophy, administration of HMB attenuates the loss in lean mass, restores muscle functionality and recovers the shrinkage of the fibers. By using L6 muscle cells in culture, we have shown that the effects of HMB were mediated by modulation of the formation of autophagosomes and of the expression of LC3, p62 and Bnip3, all belonging to the autophagic-lysosomal system. Moreover, we found that HMB prevents proteolysis by controlling the proteasome pathway. The effects of HMB were mediated by an increase in PI3K/Akt signaling and the phosphorylation of FoxO3a.

### HMB protects against DEX-induced muscle atrophy in a rat model

**Fig 1:**
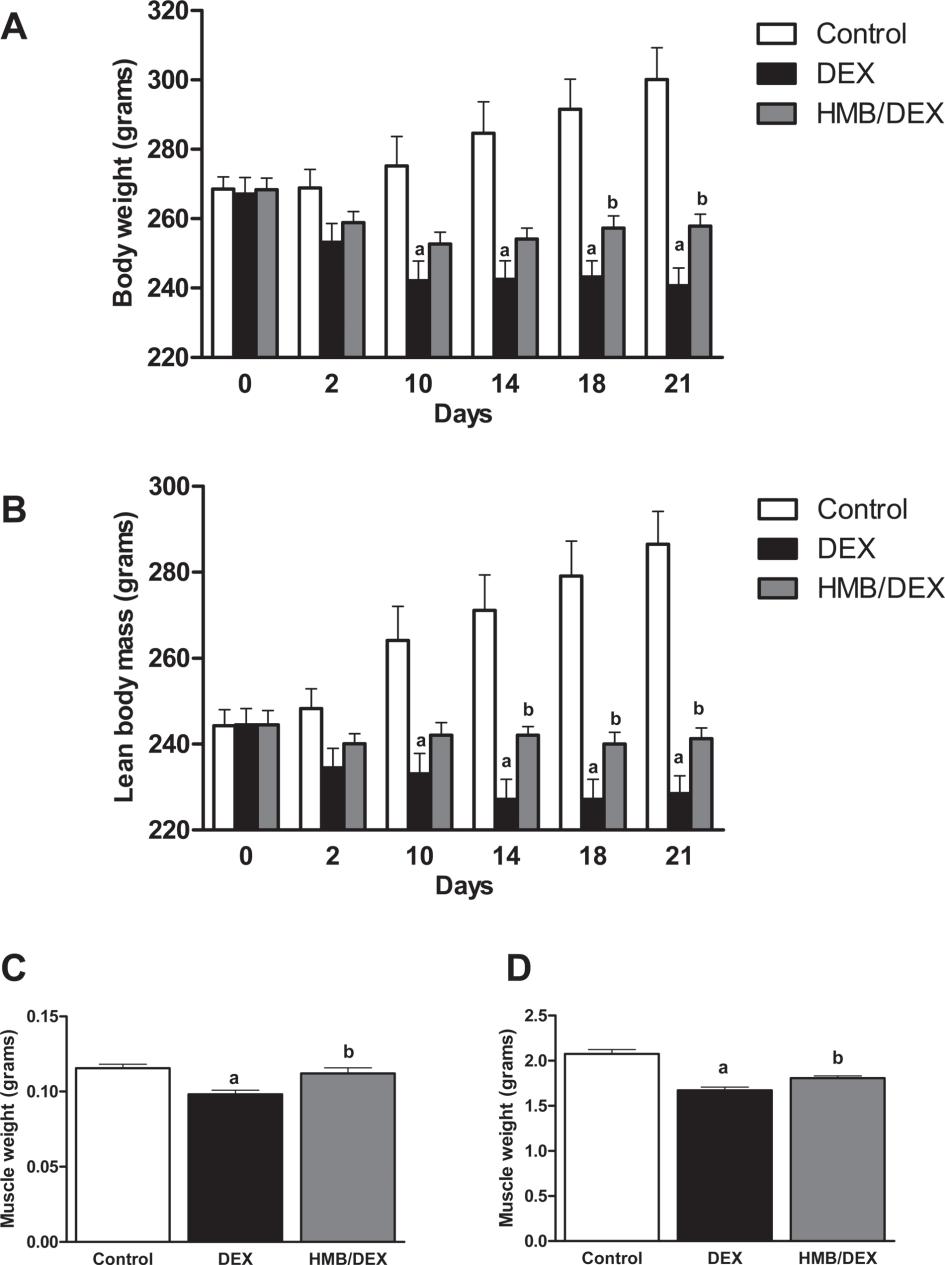
Effects of HMB supplementation on body weight, lean body mass and muscle weight in DEX-treated rats. **(A)** Body weight and **(B)** lean body mass analysis of each group (Control, DEX and HMB/DEX). Day 0 was considered as the day of initiation of dexamethasone treatment. Wet weight (grams) of soleus **(C)** and gastrocnemius **(D)** for each group at the end of the experiment. Values are expressed as mean ± SEM (n = 7). ^a^p<0.05 compared with Control group. ^b^p<0.05 compared with DEX group.

To study the effects of HMB on a DEX-induced muscle wasting model, rats were treated either with DEX (0.1 mg/kg) or DEX plus Ca-HMB (320 mg/kg) for 21 days. Administration of HMB was initiated 7 days prior to DEX treatment as a preventive approach. Our results showed that DEX caused a progressive loss of body weight compared to control rats (Fig. 1A) while administration of HMB to DEX rats significantly attenuated body weight loss from day 18.

To analyze whether the changes in body weight were due to changes in lean mass, Echo MRI determinations were carried out. As shown in Fig. 1B, the control group gained lean mass whereas DEX induced a gradual decline throughout the treatment. Co-administration of HMB attenuated the loss of lean mass, having significant effects at day 14. The amelioration in body weight and lean mass produced by HMB was parallel to the changes in wet weight of soleus (Fig. 1C) and gastrocnemius (Fig. 1D) muscles measured at the end of the experiment.

**Fig 2:**
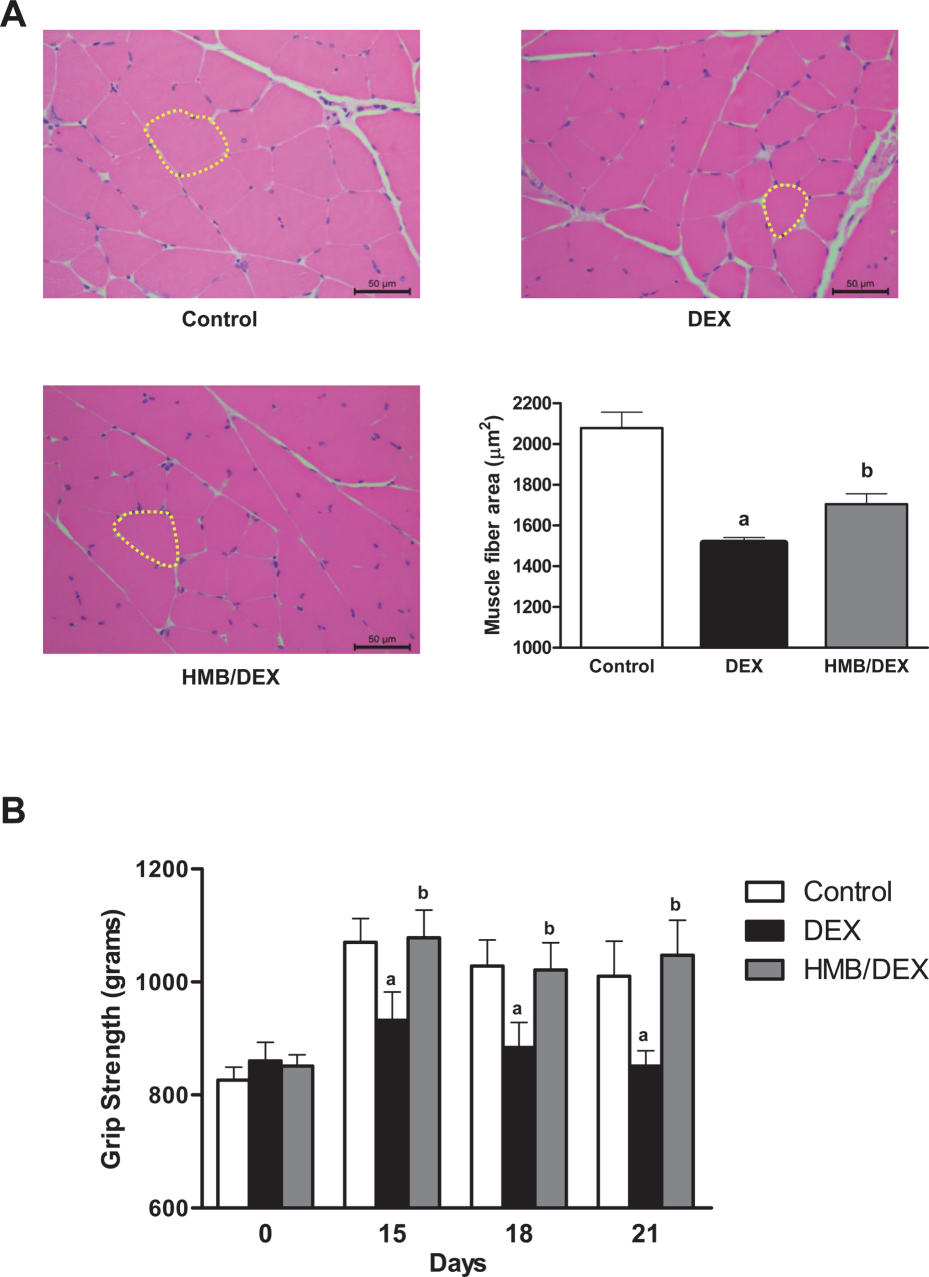
Effects of HMB supplementation on morphometric analysis and hind limb strength in DEX-treated rats. **(A)** Muscle fiber cross-sectional area (μm^2^). Measurements were made 21 days post treatment. **(B)** Hind limb strength in animals submitted to saline, DEX or HMB/DEX. Values are expressed as mean ± SEM (n = 7). ^a^p<0.05 compared with Control group. ^b^p<0.05 compared with DEX group.

To gain further insights into the effects of HMB on skeletal muscle under DEX treatment, the cross-sectional areas of gastrocnemius muscle fibers were determined. DEX promoted a reduction in the cross-sectional area (Fig. 2A) compared with the control group while co-treatment with HMB demonstrated a significant attenuation in the loss of cross-sectional area.

Finally, to determine if the changes in lean mass and fiber cross section correlated with muscle functionality, a grip strength test was conducted throughout the timecourse of the experiment. As shown in Fig. 2B, treatment with DEX decreased muscle functionality starting from day 15, whereas co-administration of HMB with DEX completely normalized muscle performance.

Our results suggest that significant HMB protection was provided after at least 14–18 days of treatment (Fig. 1). These results are in agreement with those of Hao et al. [25], who reported that 28 days HMB supplementation was able to improve skeletal muscle functionality in a model of fiber atrophy in aged rats. In contrast, a recent study [26] has reported that HMB supplementation for 7 days did not attenuate cross sectional area in muscle fibers or improved skeletal muscle performance in rats under DEX treatment. This discrepancy is probably due to the differences in the length and nature of the treatment (7 days versus 21 days). In fact, our results for body weight, lean body mass and muscle functionality show that at least 15–18 days were necessary to produce a significant recovery from DEX treatment. Moreover, in our experimental design HMB was given in drinking water *ad libitum* instead of in a single oral dose, ensuring that sustained circulating plasma concentrations of HMB were maintained during the experiment.

### Effects of HMB on protein turnover in DEX-treated myotubes

Having established the positive effects of HMB on the DEX rat model of muscle atrophy, an L6 myotube culture was used to elucidate the HMB molecular bases of action. L6 myotubes have been previously used as an *in vitro* system to determine the effects of HMB on protein turnover [16]. In our experiments, L6 cells were differentiated into myotubes, pre-incubated with or without 25 μM HMB and then incubated in the presence or absence of 5 μM DEX, as described in the methods section.

**Fig 3:**
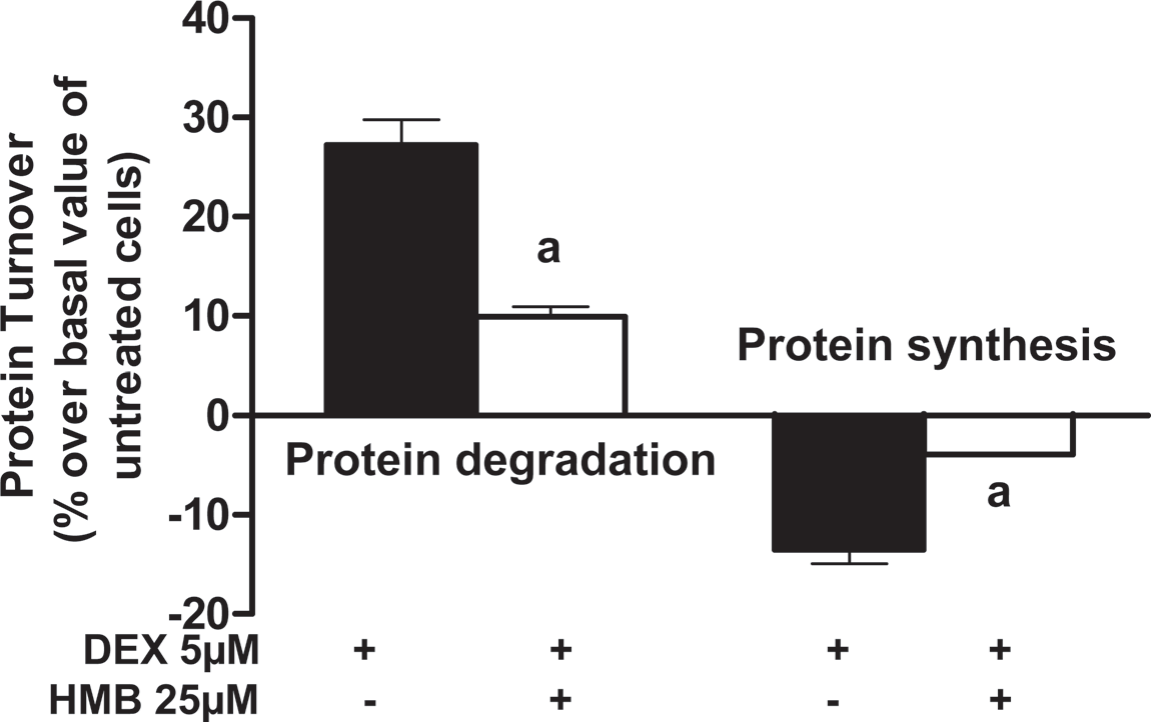
Effect of HMB on protein degradation and synthesis in rat L6 myotubes treated with DEX. Results are shown as the increase in protein degradation and the decrease in protein synthesis (%) due to the presence of DEX when compared with basal levels (non-treated cells). Results are expressed as means ± SEM (n = 8).^a^p ‹ 0.05 compared with DEX treated cells.

First, the effects of HMB on protein degradation and protein synthesis induced by DEX were analyzed. Incubation with DEX induced a significant increase in protein degradation and a decrease in protein synthesis compared with untreated L6 myotubes (Fig. 3). HMB administration to myotubes significantly decreased proteolysis induced by DEX and was also able to attenuate the decrease in protein synthesis produced by the glucocorticoid treatment. Therefore, these results validate the use of L6 myotubes incubated with DEX as a cellular model of muscle atrophy to study the effects of HMB.

### Effects of HMB on autophagy

Autophagy is a process that allows cell degradation, and, consequently, the recycling of cytosolic proteins and organelles in response to starvation. Autophagy is also responsible for the degradation of protein aggregates, which would accumulate and be toxic for the cell [27]. This process is coordinated by a battery of genes. The term autophagic flux describes autophagy as a dynamic process, that includes autophagosome formation, maduration and fusion with lysosomes with the consequent breakdown and release of macromolecules back into the cytosol [28].

One remarkable result obtained in the animal model was a reduction in the shrinkage of muscle fibers and an improvement in muscle functionality by HMB. Since DEX-induced muscle wasting has been correlated with an increase in autophagy as an altered remodeling process [29], the effects of HMB on the formation of autophagosome vesicles after DEX exposure were evaluated. The most used methods to monitor autophagic activation are based on the detection of autophagosome formation by fluorescence microscopy and the analysis of LC3 processing by western blot [30].

**Fig 4:**
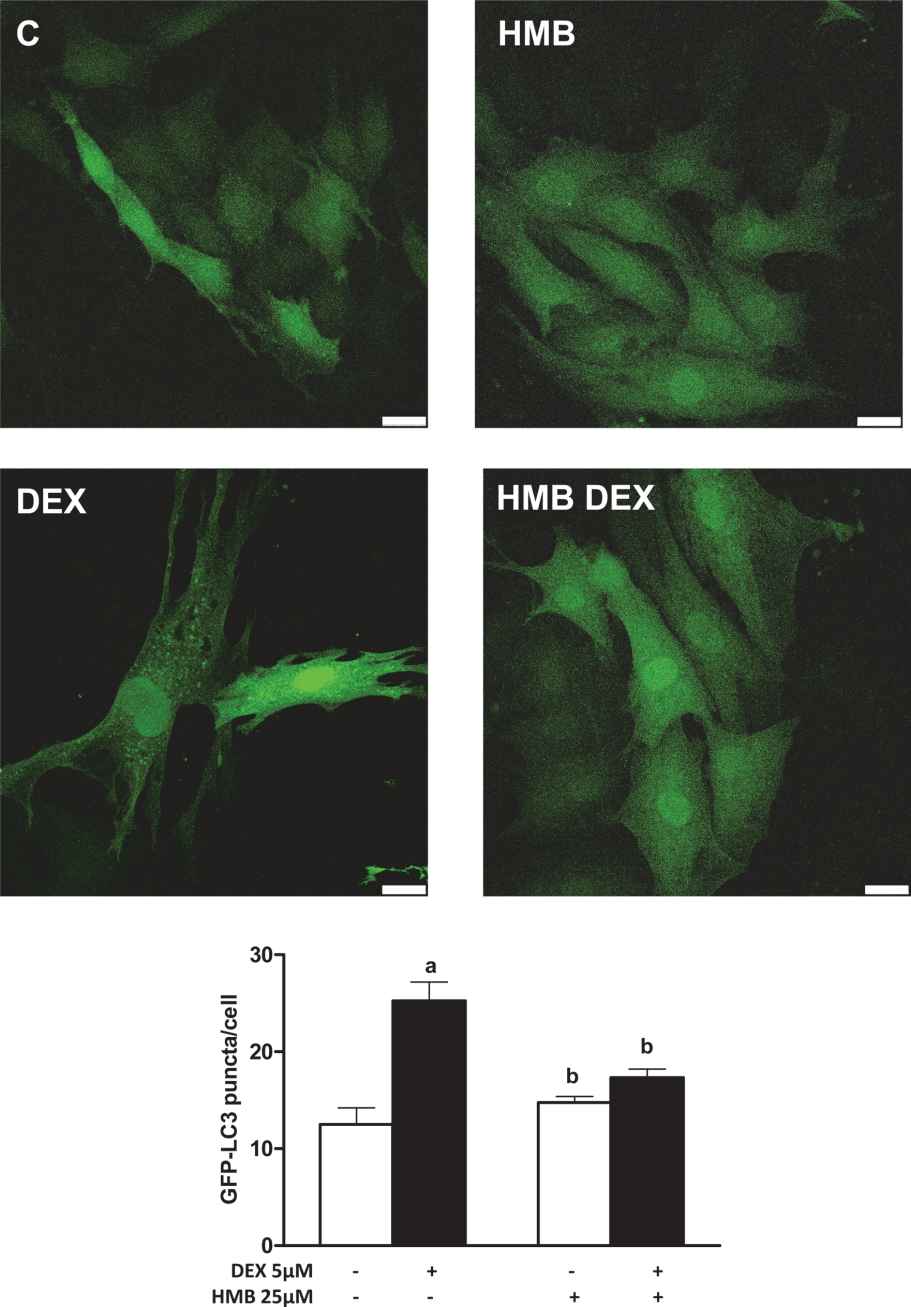
Effects of HMB supplementation on the autophagosome formation induced by DEX. Autophagosomes were quantified by counting GFP-LC3 punctae/cell of at least 4 fields per treatment. Scale bar in the microphotographs corresponds to 25 μm. Results represent means ± SEM (n = 4).^a^ p < 0.05 compared with untreated control cells; ^b^ p < 0.05 compared with DEX-treated cells.

For the analysis of autophagy, L6 cells were transfected with a GFP-LC3 plasmid and differentiated into myotubes. Next, the numbers of GFP-positive punctae/cell were counted. Our results showed that DEX treatment induced an increase in the formation of autophagosomes (Fig. 4). In contrast, pre-incubation of myotubes with HMB was able to decrease formation of GFP-autophagosomes induced by DEX. Moreover, puncta formation was not changed by HMB in the absence of DEX.

**Fig 5:**
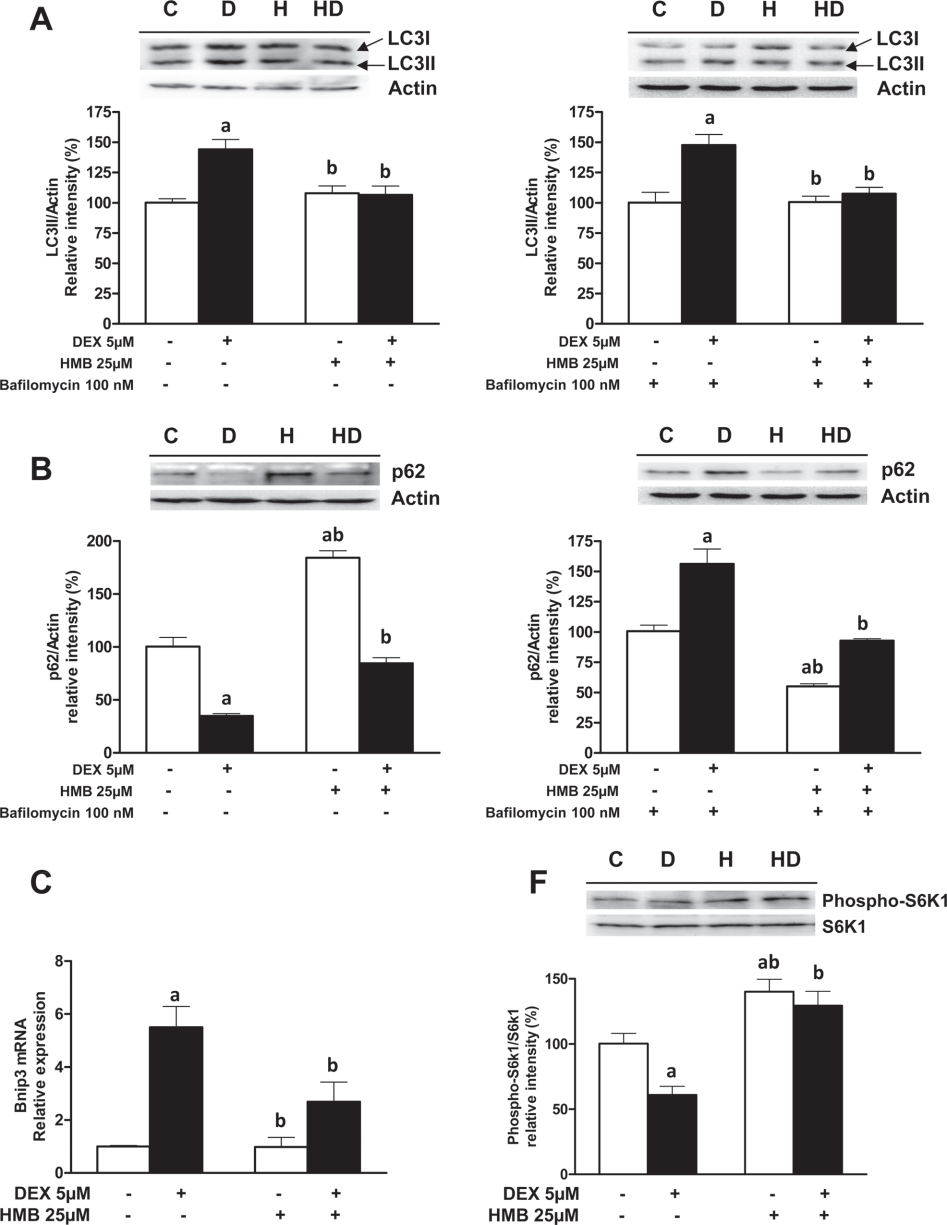
Effects of HMB supplementation on the regulation of LC3, p62, Bnip3 and S6K1 expression induced by DEX. **(A)** Immunoblotting for LC3 in absence or presence of Bafilomycin A1. Two bands are shown, corresponding to LC3-I and the lipidated form LC3-II. **(B)** Levels of p62 were evaluated by immunoblot analysis in the absence or presence of 100 nM Bafilomycin A1. **(C)** mRNA levels for Bnip3 were measured by quantitative real-time PCR analysis in samples from all the experimental groups (n = 8). **(D)** Phosphorylation level of S6K1. Signal densities from untreated cells were assigned a value of 100%. Results are expressed as means ± SEM (n = 4). ^a^ p ‹ 0.05 compared with untreated cells and ^b^ p ‹ 0.05 compared with DEX-treated cells.

The amount of LC3-II, a lipidated form of LC3, is closely related to the number of autophagosomes [30]. LC3-II is located on the inner and outer membranes and is indispensable for membrane biogenesis and closure of the membranes [31]. Once the autophagosome is mature, the LC3-II located on the outer membrane is cleaved off by Atg4B and recycled, while the LC3-II on the inner membrane is degraded by lysosomal enzymes [3]. If cells are treated with Bafilomycin A1, an autophagosome-lysosome fusion inhibitor, degradation of LC3-II is partially inhibited whereas that of LC3-I is not affected [30]. The changes in the LC3 lipidation process induced by DEX leading to the synthesis of LC3-II were normalized by HMB treatment. These results have been confirmed by the use of bafilomycin A1 (Fig. 5A). Bafilomycin prevents the lysosomal degradation of LC3-II and allows a better analysis of the autophagic process [30]. In the presence of Bafilomycin A1, an increase in the expression of LC3-II and concomitant decrease in LC3-I was observed in the DEX-treated cells. HMB was again able to normalize the amount of LC3-II.

Monitoring the degradation of p62 (SQSTM1/sequestosome-1) is an alternative method to detect the autophagic flux [3, 27]. p62 is an ubiquitin-associated protein that interacts directly with LC3-II [32]. In the absence of Bafilomycin A1, the level of p62 decreased in response to DEX treatment while pre-incubation with HMB restored its expression (Fig. 5B). It is interesting to point out that incubation with HMB alone increased p62 levels. It is described that p62 is degraded by both the autophagy and ubiquitin-proteasome systems [32] and the increase due to HMB incubation could be due to an inhibition of the proteasome. In contrast, when cells were treated with an inhibitor of autophagosome lysis, DEX promoted an increase in p62 levels that was reverted by pre-incubation with HMB. Taken together, the p62 results sustained the idea that HMB is able to counteract DEX induced autophagy.

To gain further insights into the regulation of the autophagy pathway, mRNA levels of Bnip3, as an inductor of autophagosome formation [9, 27], were measured. HMB reduced the increase in the mRNA levels of Bnip3 caused by DEX treatment (Fig. 5C).

Finally, we analyzed the phosphorylation of S6K1 as a marker not only related to protein synthesis but also to an increase in autophagy [33]. DEX treatment induced a decrease in the activation of S6K1. In contrast, pre-treatment with HMB was able not only to block the effects induced by DEX but also to significantly increase the phosphorylation of S6K1 (Fig. 5D).

Taken together, the effects of HMB on GFP-LC3 punctae, on the expression of LC3-II, p62 and Bnip3 as well as on S6k1 phosphorylation levels clearly establish a normalization of autophagic flux as a model of action for HMB on DEX-induced muscle atrophy. This is the first time that the effects of HMB on muscle wasting have been associated with normalization of autophagy, and the elucidation of the molecular bases for HMB regulation of autophagy are relevant in order to validate the potential beneficial effects of HMB on muscle wasting conditions. Furthermore, it should be remembered that the addition of HMB does not change the basal levels of autophagy of control cells and thus does not block this essential muscle process [24] under normal conditions.

### HMB decreases FoxO transcriptional activity in DEX-treated myotubes

It has been reported that Foxo3a induces the expression of autophagy-related genes [5,6,9] including LC3 and Bnip3. The role of FoxO, as a transcriptional activator, is typically inhibited by insulin [27]. In contrast, DEX acts as a FoxO activator by inhibiting the PI3K/Akt signaling pathway [7]. Since FoxO has been established as the key element for DEX-induced autophagy, we studied the effects of HMB on the phosphorylative status of FoxO3a and FoxO-dependent transcriptional activity.

**Fig 6:**
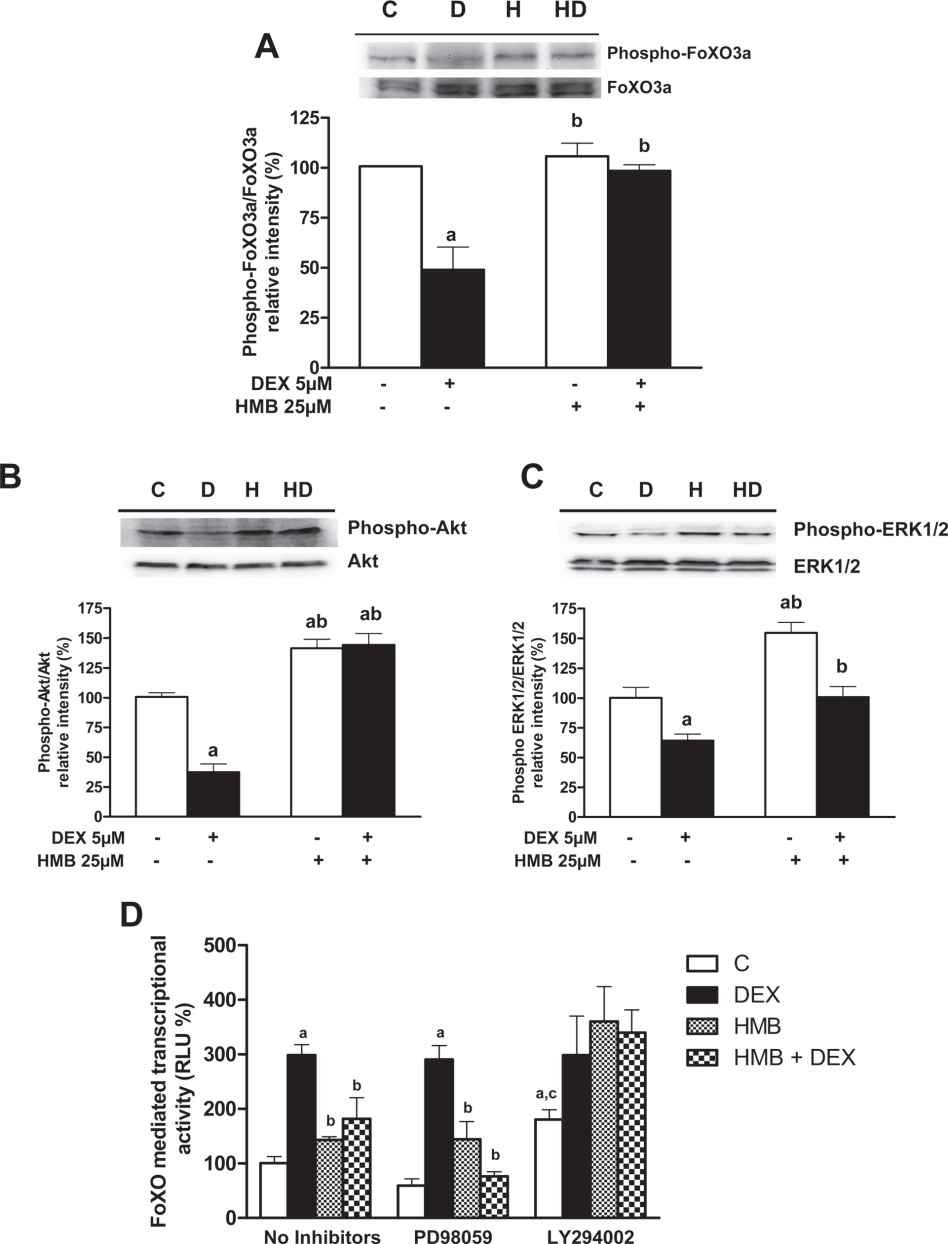
Effects of HMB on the phosphorylation of FoxO3a, Akt and ERK1/2 and on FoxO transcriptional activity in presence of DEX. Cells were lysed and total protein was immunoblotted with specific phospho- and total-antibodies against FoxO3 (**A),** Akt/PKB (**B**) or ERK1/2 **(C)**. Signal densities from untreated cells were assigned a value of 100%. Results are expressed as means ± SEM (n = 4).^a^ p ‹ 0.05 compared with untreated cells and ^b^ p ‹ 0.05 compared with DEX- treated cells. **(D)** Cells were transiently transfected with a FoxO luciferase reported plasmid and differentiated into myotubes before treatments to evaluate FoxO-dependent transcription. Cells were pre-incubated for 30 min with 10 μM PD98059 and 20 μM LY294002, then incubated with 25 μM HMB for 48 h and finally incubated with 5 μM DEX in the presence or absence of effectors. Inhibitors were maintained during the experiment. Results are expressed as means ± SEM (n = 6). ^a^p ‹ 0.05 compared with control cells in the presence of each inhibitor, ^b^p ‹ 0.05 compared with DEX- treated cells in the presence of each inhibitor and ^c^p ‹ 0.05 compared with control cells in the absence of inhibitors.

DEX treatment decreased FoxO3a at Thr32 phosphorylation (Fig. 6A) with a concomitant increase in FoxO-dependent transcriptional activity (Fig. 6D). Treatment with HMB normalized FoxO3a phosphorylation and consequently inhibited FoxO transcriptional activity. It should be noted that HMB alone was unable to significantly change the phosphorylative status of FoxO or its transcriptional activity compared with control cells. This result is in agreement with the levels of autophagy obtained in control or HMB-treated cells.

FoxO transcriptional activity is mainly regulated by phosphorylation. Protein kinases such as Akt directly phosphorylate FoxO factors, resulting in sequestration at the cytosol and repression of their transcriptional activity [2,5,6,7,9,27]. The three key phosphorylation sites in FoxO3A are Thr32, Ser253, and Ser315, corresponding with a consensus sequence for Akt phosphorylation [34]. On the other hand, the effects of DEX on muscle wasting have been ascribed to a hypo-phosphorylation of FoxO and concomitant migration to the nucleus through inhibition of the corresponding kinases [6,7].

Previous experiments have suggested that the effects of HMB on muscle could be mediated via the PI3K/Akt and MAPK/ERK pathways [13,14]. Consequently, we assayed the effects of HMB in the presence or absence of DEX on the phosphorylation status of PKB/Akt and ERK1/2. DEX significantly reduced the phosphorylation of PKB/Akt and ERK1/2 while HMB significantly stimulated the phosphorylation of both kinases. When HMB-pretreated cells were incubated with DEX, the phosphorylative status of PKB/Akt remained higher than that of control cells (Fig. 6B) while the activation of ERK1/2 was similar to that observed in the untreated cells (Fig. 6C). Therefore, both pathways were restored by HMB treatment in the DEX cells.

To assess the involvement of either PKB/Akt or ERK1/2 on the HMB-mediated modulation of FoxO transcriptional activity, L6 myotubes transfected with the FoxO reporter were pre-incubated with inhibitors of PKB/Akt (20 μM LY294002) or ERK1/2 (10 μM PD98059) mediated signaling prior to treatment with HMB and DEX (Fig. 6D). While the inhibition of the ERK1/2 signaling pathway did not change either the effects of DEX or HMB on FoxO-dependent transcriptional activity, the blockade of Akt signaling induced an increase in FoxO activity in untreated cells as expected [7] and blocked the effects of HMB in DEX-treated cells. Thus, the effect of HMB in reducing the activity of FoxO in DEX-treated cells was due to a re-activation of PKB/Akt signaling and was independent of ERK 1/2 pathway.

Finally, to illustrate the dependence of the Akt vs mTOR in the HMB-induced suppression of autophagy, myotubes were treated with DEX or HMB + DEX in the presence of LY294002, an Akt inhibitor, or rapamycin, a mTOR inhibitor and the effects on LC3-II expression as a marker of autophagy were assayed. Immunoblots of LC3-II showed that the HMB-induced suppression of autophagy was strongly dependent of the Akt signaling compared with the mTOR pathway (S1 Fig.).

### HMB pre-treatment decreases ubiquitin promoter transcriptional activity and atrogenes expression in DEX-treated myotubes

Most studies of the beneficial effects of HMB in preventing proteolysis in muscle are based on the total or partial decrease in the expression of ubiquitin ligases [16, 35] as components of the proteasome system. However, the effects of HMB on the transcription of the ubiquitin gene have not been reported.

**Fig 7:**
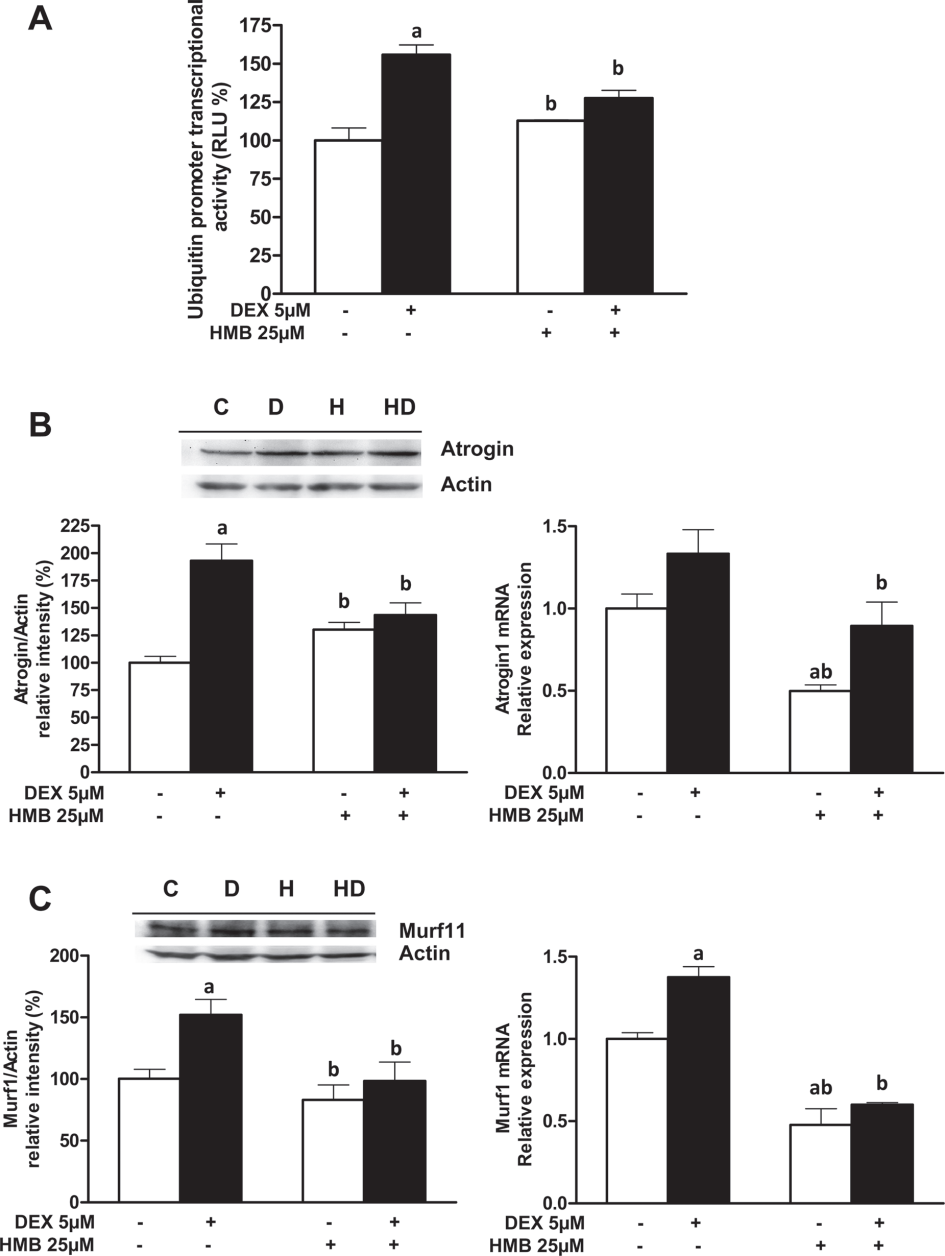
Effects of HMB on Ub-C promoter activity and expression of Atrogin-1 and MuRF1 induced by DEX. **(A)** Cells were transiently transfected with an UbC-luciferase reported plasmid and differentiated into myotubes before treatments to evaluate UbC gene transcription (n = 6). Western blots for Atrogin-1 **(B)** and MuRF1 **(D)**. Densitometric quantification of protein abundance using actin as a reference control is shown. Signal densities from untreated cells were assigned a value of 100% (n = 4). mRNA levels for Atrogin-1 **(C)** and MuRF1 **(E)** were measured in samples from all the experimental groups (n = 8). Results are expressed as means ± SEM. ^a^p ‹ 0.05 compared with untreated cells and ^b^p ‹ 0.05 compared with DEX-treated cells.

An *in silico* analysis of the rat ubiquitin promoter using PROMO software [36] showed the existence of two binding sites for FoxO3a (at positions -199 and -381). Since, in muscle atrophy, the Ub-C mRNA increased more than other ubiquitin mRNAs (ie UbA and UbB) [37] we used a reporter system based on the rat Ub-C promoter fused to a luciferase gene as a sensitive marker for analyzing general activation of the Ub-proteasome. Furthermore, atrogin-1 and MuRF1 (E3-ubiquitin ligases) are, at least in part, under the control of FoxO3a [29]. Therefore, the effects of DEX and HMB on the transcription of the ubiquitin gene (Fig. 7A) and the expression and mRNA levels of both ubiquitin ligases have been assayed (Fig. 7B- 7E).

Our results showed that DEX treatment induced an increase in Ub-C promoter activity while pre-treatment with HMB was able to significantly counteract the effects of DEX on Ub-C promoter activity (Fig. 7A). Furthermore, pre-incubation with HMB significantly reduced the DEX-induced expression and mRNA levels of Atrogin-1 (Fig. 7B and 7C) and MuRF1 (Fig. 7D and 7E). In the absence of DEX, HMB did not modify the basal amount of either of the ubiquitin ligases. Our results indicate that HMB is able to decrease ubiquitin-dependent protein breakdown and therefore explain the increase in p62 levels in the presence of HMB [32].

## Conclusions

HMB exerts protective effects against muscle atrophy promoted by DEX *in vivo* and *in vitro*. Firstly, *in vivo* experiments demonstrated that co-administration of HMB attenuates loss of lean mass and the shrinkage of the fibers, and restoring muscle functionality. Secondly, we demonstrated that the preventive effects of HMB in an *in vitro* model of muscle atrophy were mainly mediated by modulation of the autophagy-lysosomal system. The key element in HMB normalization of autophagy is the phosphorylation of FoxO3a as a consequence of an increase in PI3K/Akt signaling in the cytosol. Finally, and given that FoxO also regulates the transcription of genes belonging to the proteasomal system, HMB prevents proteolysis by controlling the activity of ubiquitin and the expression of ubiquitin ligases.

## Supporting Information

S1 Fig Dependence of Akt vs mTOR in the HMB-induced suppression of autophagy. Cells were pre-incubated for 30 min with 20 μM LY294002 and 20 nM rapamycin, then incubated with 25 μM HMB for 48 h and finally incubated with 5 μM DEX in the presence or absence of effectors. Inhibitors were maintained during the experiment. Cells were lysed and total protein was immunoblotted with LC3 antibody. Results are expressed as means ± SEM (n = 4).^a^ p ‹ 0.05 compared with untreated cells and ^b^ p ‹ 0.05 compared with DEX- treated cells.
(TIF)
