# Supplementary figures and images for: β-Hydroxy-β-Methylbutyrate (HMB) Normalizes Dexamethasone-Induced Autophagy-Lysosomal Pathway in Skeletal Muscle
Source: PLoS One. 2015 Feb 6;10(2):e0117520. doi: 10.1371/journal.pone.0117520 (PMC4319954; doi:10.1371/journal.pone.0117520)

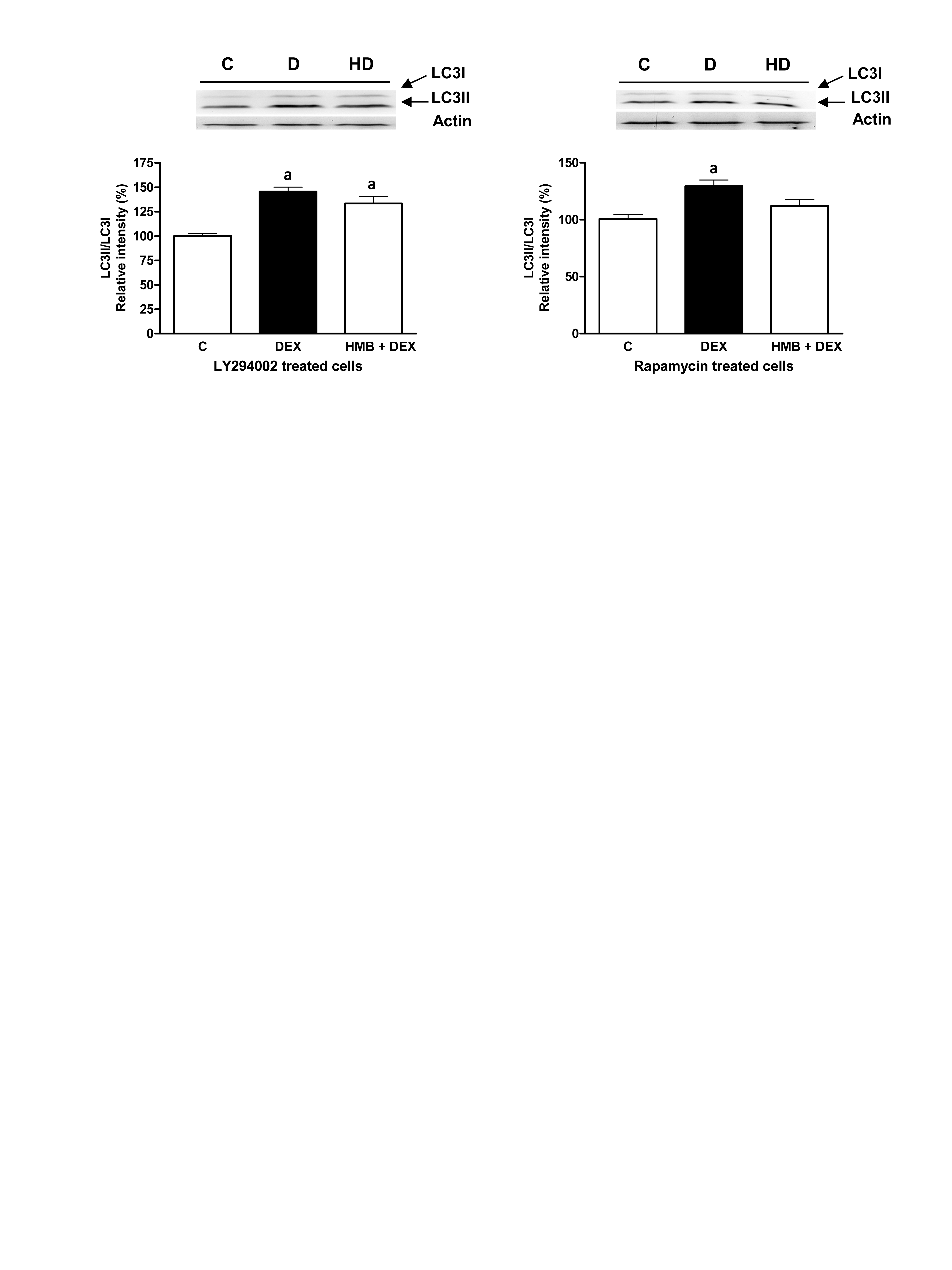

Supplement: S1 Fig — Cells were pre-incubated for 30 min with 20 μM LY294002 and 20 nM rapamycin, then incubated with 25 μM HMB for 48 h and finally incubated with 5 μM DEX in the presence or absence of effectors. Inhibitors were maintained during the experiment. Cells were lysed and total protein was immunoblotted with LC3 antibody. Results are expressed as means ± SEM (n = 4).a p ‹ 0.05 compared with untreated cells and b p ‹ 0.05 compared with DEX- treated cells. (TIF) [file pone.0117520.s001.tif]
